# Editorial: Context in Communication: A Cognitive View

**DOI:** 10.3389/fpsyg.2017.00115

**Published:** 2017-02-06

**Authors:** Gabriella Airenti, Alessio Plebe

**Affiliations:** ^1^Department of Psychology, Center for Cognitive Science, University of TorinoTorino, Italy; ^2^Department of Cognitive Science, University of MessinaMessina, Italy

**Keywords:** context, pragmatics, communication, common ground, theory of mind (ToM)

Context is a controversial concept. Research in philosophy of language, linguistics and cognitive science has shown that the communicative content of an utterance is not limited to the conventional content of what is said. The notion of context has been introduced in semantics and has assumed a central role in language studies with the pragmatic turn that has shifted the focus from meaning to speaker's meaning, a change of paradigm that can be traced back to Wittgenstein's conception of language use (Wittgenstein, 1953) and to the work of philosophers of language like Austin (1962), Grice (1975, 1978), and Searle (1969). In this framework pragmatics deals with the intentional aspects of language use. The notion of context is then no more restricted to the interpretation of indexicals and demonstratives (Kaplan, 1989). More generally, it applies to what is presupposed as common ground among the participants in a conversation (Stalnaker, 2002, 2014).

From a cognitive perspective communication is an inferential process based on mental states and shared knowledge (Clark, 1996). What contributes to interpret a communicative act beyond the spoken words may, broadly speaking, be included. Intuitively, context is the background for comprehension, what makes communication possible. This is a critical point. In fact, context both is an inescapable concept in the study of communication and eludes univocal definition. There is no one context but many.

In launching this Research Topic we did not expect to find a final definition or to have the last say. We were interested in singling out the present lines of research in this field. The papers we have collected attack the problem from different perspectives and using different research methodologies.

The paper by Faber and León-Araúz is aimed at, if not final, a comprehensive and detailed definition of context. They propose a taxonomy based on scope: local, spanning typically five items before or after the term occurrence; and global, such as a whole text or all that goes beyond the text such as the communicative situation. They apply this distinction to syntax, semantics, and pragmatics even if, as they note, at this level the boundaries are fuzzy. The challenging enterprise of detailing what context is, becomes mandatory in formalizing specialized knowledge resources, but the results shed light on the structure of context in general language.

On the way of clarifying what context constitutively is, García-Carpintero addresses Stalnaker's notion of context as common ground, mentioned above, showing certain weaknesses. The Stalnakerian view of common ground as sets of propositions reveals unsatisfying in cases of expressions with rich illocutionary features. The most convincing cases are those of slurs and pejoratives, where attempts to flatten the content into declarative form, will deprive context of important dimensions of expressive meaning. Therefore, context, in addition to sets of propositions, should be extended to include shared propositional commitments. Although the case of pejoratives and slurs is the most convincing, the requirement for shared commitments appears in other cases examined by Garcia-Carpintero as well: directives, questions, predicates of taste, pretense.

Notably, the set of shared commitments proposed by Garcia-Carpintero includes aspects of the emotional state of the speaker. A step further inside the personal and interpersonal spheres is taken by Marques, investigating predicates of personal taste, aesthetic or moral values. A well known drawback afflicting contextual explanations is disagreement. If two conflicting judgments can be explained by simply augmenting the original sentences with propositions about the context of the two speakers, disagreement should disappear. Marques argues for contextualism, suggesting that disagreement can be addressed by taking into account differences in non-doxastic attitudes, and is enhanced by the evolutionary reinforcement of certain personal dispositions in social coordination.

The main contender to the contextualist strategy defended by Marques is relativism, which is contrasted with expressivism in the paper by Frápolli and Villanueva. The idea is that there are two main ways to accommodate context dependence, by what they call *building-block* or *organic models*. The former, that gives prominence to the principle of compositionality over the principle of context, is proper to relativism, while the latter, that privileges context over compositionality, belongs to expressivism.

While in the group of papers described so far, the main perspective under which context is studied is semantic, enriched with insights on mental phenomena, in the next group the cognitive perspective prevails, asking questions about how context is structured and accessed in the mind. Mazzone builds upon one of the most developed theories in cognitive pragmatics, Relevance Theory (Sperber and Wilson, 1986) and discusses how this theory succeeds in explaining the way relevant context is constructed during utterance understanding. He identifies a weakness in spelling out the mechanisms in place during the process of selecting the context, which, suggests Mazzone, can instead be identified in the combination of a bottom-up activation of schemata, especially goal-directed schemata, with a top-down activation of contextual information. This sort of mechanism is supported by what is currently known about the hierarchical structure of the frontal cortex.

Relevance Theory is the starting assumption also for Attardo, in the search for a satisfactory context to explain utterances. He stresses how the exploration of relevance is largely abductive in nature, and remarks that the derivation of context requires additional mechanisms that counteract the expansive tendencies of relevance and abduction. Such bonding mechanisms, argues Attardo, can be construed under the principles of *satisfaction* and *charity*.

Paradigmatic in a cognitive perspective on context is the discussion about the so-called Theory of Mind (ToM), the set of skills that allow to attribute beliefs, goals, and percepts to other people: how essential is this ability in constructing the context necessary to understand utterances? The two contributions by Kissine and Cummings provide two contrasting answers. For Kissine there are grades of interpretative strategies to derive relevant implicatures of an utterance, and the lower levels, like the *egocentric relevance*, do not require any ToM. For Cummings utterance interpretation is highly dependent on attributing cognitive and affective mental states to the minds of language users, and she proposes that for the purpose of context derivation the best notion of ToM should encompass the rational, intentional, holistic character of interpretation. Both papers draw on studies with ASD (Autism Spectrum Disorder) subjects to support their arguments. Kissine reports of subjects with ASD able to correctly discriminate between “ironical” and “literal” interpretations. Cummings reports clinical cases where ASD subjects exhibit deficits covering the three cornerstones of ToM she identified: rationality, intentionality, and holism.

Airenti investigates young children's ability to produce and understand different forms of humor. In particular she focuses on teasing, a form of humor already present in preverbal infants that is also considered a typical feature of irony. She proposes that the acquisition of specific communicative contexts enable children to engage in humorous interactions before they possess the capacity to analyze them in the terms afforded by a full-fledged ToM.

In addition to increase our understanding, the cognitive perspective on context has important practical implications, as in the divergent interpretations of numeric quantities reported by Mandel. Subjects tend to assume large numerical quantities not as exact values, rather adopting a lower-bound *at least* or an upper-bound *at most* interpretation, depending on the context.

Several papers fall within the domain of experimental pragmatics.

Filippi et al. explore the role of prosodic cues in word learning. In natural situations learners have to identify words within a sequence of sounds and to relate them to specific referents extracted by the visual scene. Developmental research has suggested that adults' use of exaggerated pitch might direct infants' attention to specific elements in the context and guide learning. In their study the authors show that also adults exposed to an artificial language in different experimental conditions exploit pitch enhancement as a pragmatic cue.

The role of intonation employed as an indicator of focus in pragmatic interpretation is treated in Cummins and Rohde. In Gricean pragmatics the interpretation of an utterance is based on the relation between what has been said and the potential utterances that would have been relevant to the current discourse purpose, had it been uttered. This set of relevant alternatives is defined in the notion of Question Under Discussion (Roberts, 1996/2012). The three experiments reported in this study showed that hearers used the intonation as an indication of which QUD is currently in play in the interpretation of scalar implicatures, presuppositions, and coreference.

Domaneschi et al. maintain that for the analysis of context an important role is played by cognitive load. In fact, cognitive effort might have an effect on which presuppositions are activated. In their study they show this effect with presupposition selection in conditional sentences with a trigger in the consequent. The effect of cognitive effort in interpreting communicative utterances involving pragmatic enrichment is also the subject of Janssens and Schaeken's paper. However, their study showed no influence of the working memory load on the performance in the task of inferring the implicatures from *but, so* and *nevertheless*. They also found that a major role in interpretation is played by the content of the arguments suggesting that context and content are fundamental in the interpretation process.

In their paper Dupuy et al. discuss how the context affects the interpretation of scalar implicatures. In particular, they focus on the pragmatic interpretation of *some*. They test two factors, the existence of factual information that facilitates the computation of pragmatic interpretations in the context, i.e., the cardinality of the domain of quantification, and the fact that the context makes the difference between the semantic and the pragmatic interpretations relevant. Their results suggest that the main factor that enhances pragmatic interpretation is the relevance of the contrast that in turn increases the salience of the cardinality.

Two papers use event-related potential (ERP) electrophysiological technique to analyze the role of context in the comprehension of two important pragmatic phenomena, metaphor and referential ambiguity. Bambini et al. conducted two experiments in which EEG activity was recorded when participants were presented with metaphors in two different context situations, a minimal vs. a supportive context. Their results suggest the presence of two dissociable ERP signatures in the processing of metaphors. In fact, the N400 effect was visible only in minimal context, whereas the P600 was visible both in the absence and in the presence of contextual cues. From these data the authors argue that linguistic context reduces the effort in retrieving lexical aspects of metaphors but does not suppress later pragmatic interpretation efforts needed in order to derive the speaker's intended meaning. Jiang and Zhou investigate how a comprehender resolves referential ambiguity in a conversation by using information concerning the social status of communicators in the context, and how empathic sensitivity to the social status information modulates ambiguity perception and the underlying neural activity. Electrophysiologically, they show the existence of differential neurocognitive processes underlying ambiguity resolution with different contextual cues.

Two papers analyze communication in context as a diagnostic and clinical resource.

Arcara and Bambini propose a test (APACS) to evaluate pragmatic abilities in clinical populations with acquired communicative deficits, ranging from schizophrenia to neurodegenerative diseases. The test consists of six tasks devoted to assess different pragmatic abilities in the domains of discourse and nonliteral communication. Their assumption is that while globally depending on context, different pragmatic aspects might involve different cognitive skills.

Stahl and Van Lancker Sidtis analyze the contribution of formulaic expressions in clinical rehabilitation from speech and language disorders after stroke. For these patients formulaic expressions frequently remain one of the few resources available for communication. Therapy may support them in including these expressions within language games, i.e., communicative exchanges based on turn-taking. In this way the conversational context allows patients to exploit their residual resources in order to reestablish social interactions.

Edwards deals with an extreme case of communication reporting her fieldwork with a community of deaf-blind people in Seattle. Edwards via the analysis of interactional sequences and subjects' metapragmatic commentary shows how deaf-blind people use tactile-kinesthetic channels to overcome the difficulty to converge on objects of reference. She discusses two mechanisms that can account for this process: embedding in the social field and deictic integration. She argues that together they yield a deictic system set to retrieve a restricted range of values from the extra-linguistic context, thereby attenuating the cognitive demands of intention attribution.

In summary, this research topic is a sampling of innovative efforts to address challenging issues on context, involving complex questions spanning from brain processes to social interactions and pragmatics. This sampling witnesses a growing, vibrant community of researchers attempting to integrate the knowledge, the methods, and the theory-building tools from philosophy of language, linguistics, cognitive science, and cognitive neuroscience.

## Author contributions

Both authors contributed to the editorial and approved it.

### Conflict of interest statement

The authors declare that the research was conducted in the absence of any commercial or financial relationships that could be construed as a potential conflict of interest.
